# iTBS-Induced LTP-Like Plasticity Parallels Oscillatory Activity Changes in the Primary Sensory and Motor Areas of Macaque Monkeys

**DOI:** 10.1371/journal.pone.0112504

**Published:** 2014-11-10

**Authors:** Odysseas Papazachariadis, Vittorio Dante, Paul F. M. J. Verschure, Paolo Del Giudice, Stefano Ferraina

**Affiliations:** 1 Department Physiology & Pharmacology, Sapienza University Rome, Rome, Italy; 2 Istituto Superiore di Sanità (ISS), Rome, Italy; 3 Laboratory for Synthetic, Perceptive, Emotive and Cognitive Systems, Center of Autonomous Systems and Neurorobotics, ICREA-Universitat Pompeu Fabra, Barcelona, Spain; 4 INFN, Rome, Italy; University of Toronto, Canada

## Abstract

Recently, neuromodulation techniques based on the use of repetitive transcranial magnetic stimulation (rTMS) have been proposed as a non-invasive and efficient method to induce *in vivo* long-term potentiation (LTP)-like aftereffects. However, the exact impact of rTMS-induced perturbations on the dynamics of neuronal population activity is not well understood. Here, in two monkeys, we examine changes in the oscillatory activity of the sensorimotor cortex following an intermittent theta burst stimulation (iTBS) protocol. We first probed iTBS modulatory effects by testing the iTBS-induced facilitation of somatosensory evoked potentials (SEP). Then, we examined the frequency information of the electrocorticographic signal, obtained using a custom-made miniaturised multi-electrode array for electrocorticography, after real or sham iTBS. We observed that iTBS induced facilitation of SEPs and influenced spectral components of the signal, in both animals. The latter effect was more prominent on the θ band (4–8 Hz) and the high γ band (55–90 Hz), de-potentiated and potentiated respectively. We additionally found that the multi-electrode array uniformity of β (13–26 Hz) and high γ bands were also afflicted by iTBS. Our study suggests that enhanced cortical excitability promoted by iTBS parallels a dynamic reorganisation of the interested neural network. The effect in the γ band suggests a transient local modulation, possibly at the level of synaptic strength in interneurons. The effect in the θ band suggests the disruption of temporal coordination on larger spatial scales.

## Introduction

At the basis of the ability of the nervous system to generate adaptive behaviour lie mechanisms of synaptic plasticity such as Long-Term Potentiation (LTP). A clear link between LTP-inducing protocols and the oscillatory activity of neural populations has been reported first in slice preparation and later at a macroscopic level, showing the involvement of θ and γ rhythms in LTP [1], [2], [3], [4], [5], [6], [7], [8]. Recently, a similar phenomenon has been observed at a macroscopic level, commonly referred to as LTP-like conditioning. LTP-like conditioning refers mainly to aftereffects of repetitive transcranial magnetic stimulation (rTMS) [9]. Applying rTMS over a cortical area transiently enhances or diminishes the excitability of the area, often with behavioural correlates [9], [10]. So far, in vivo evidence of induced oscillatory changes after LTP-like conditioning in a neocortical interconnected neural population is scattered and controversial [11], [12], [13], [14].

A promising methodology to directly characterize stimulation-induced brain response at a cortical level is the combination of TMS with electroencephalography (EEG) [16]. By combining TMS with EEG, we can directly and non-invasively stimulate a cortical area and measure the effects produced by this perturbation both in amplitude and in frequency domains [15], [16]. By doing so it has been discovered that single pulse TMS induces short lasting field potential modulations of the neural activity while high-intensity rTMS pulses, induce modulations that last several minutes [17], [18], [19]. Though neural oscillatory activity is often related to cortical excitability [17], [20], [21], the relationship between LTP-like induced long-term excitability modulations and continuous neural rhythmic activity remains open.

Intermittent theta burst stimulation (iTBS) constitutes a non-invasive recently developed rTMS stimulation protocol able to induce lasting aftereffects commonly attributed to local LTP-like plasticity mechanisms [10]. The iTBS paradigm consists of repetitive, low intensity TMS pulses, unable to generate a Motor Evoked Potential (MEP) when applied over the primary motor cortex (M_1_). iTBS over both the primary somatosensory cortex (S_1_) or M_1_ leads to an increase in cortical excitability that corresponds to increased-amplitude somatosensory evoked potentials (SEP) [22], [23] and MEP [10] respectively, for approximately 30 minutes.

Still, in order to study subtle rhythmic modulations in continuous recordings a more sensitive technique than EEG is needed. Electrocorticography (ECoG) [24], [25] has the advantage of having a higher signal to noise ratio over EEG. Additionally, due to the lack of the skull barrier, high frequencies are less attenuated than in EEG studies [26] and recordings can be made from smaller electrodes with denser distribution, thus finely isolating neural populations and delivering greater spatial resolution. In humans, ECoG recordings are applied almost exclusively pre-surgically in epilepsy patients in order to better define the surgical site. As a consequence, the advantage is nulled by the unnecessary risk of applying iTBS over potential epileptic sites and would not be ethically justified [27], [28].

We used a monkey model to investigate iTBS-induced aftereffects at the mesoscopic level in the hand region of both S_1_ and M_1_, a highly interconnected network [29]. We addressed a relatively simple issue: whether and in what way does the network dynamics change between two states, an iTBS-conditioned and an unconditioned state.

## Materials and Methods

We used an iTBS protocol to stimulate the sensorimotor area in a non-human primate model and recorded induced oscillatory activity at high spatial and temporal resolution using a custom-made ECoG array. Stimulation intensity was chosen based on MEP evoked using single pulse TMS during preliminary tests. SEP modulation was evaluated during conditioning protocols to probe the effect of iTBS and confirm that rhythm modulations were attributable to iTBS after-effects.

### Animal model and Ethics Statement

**Figure 1:**
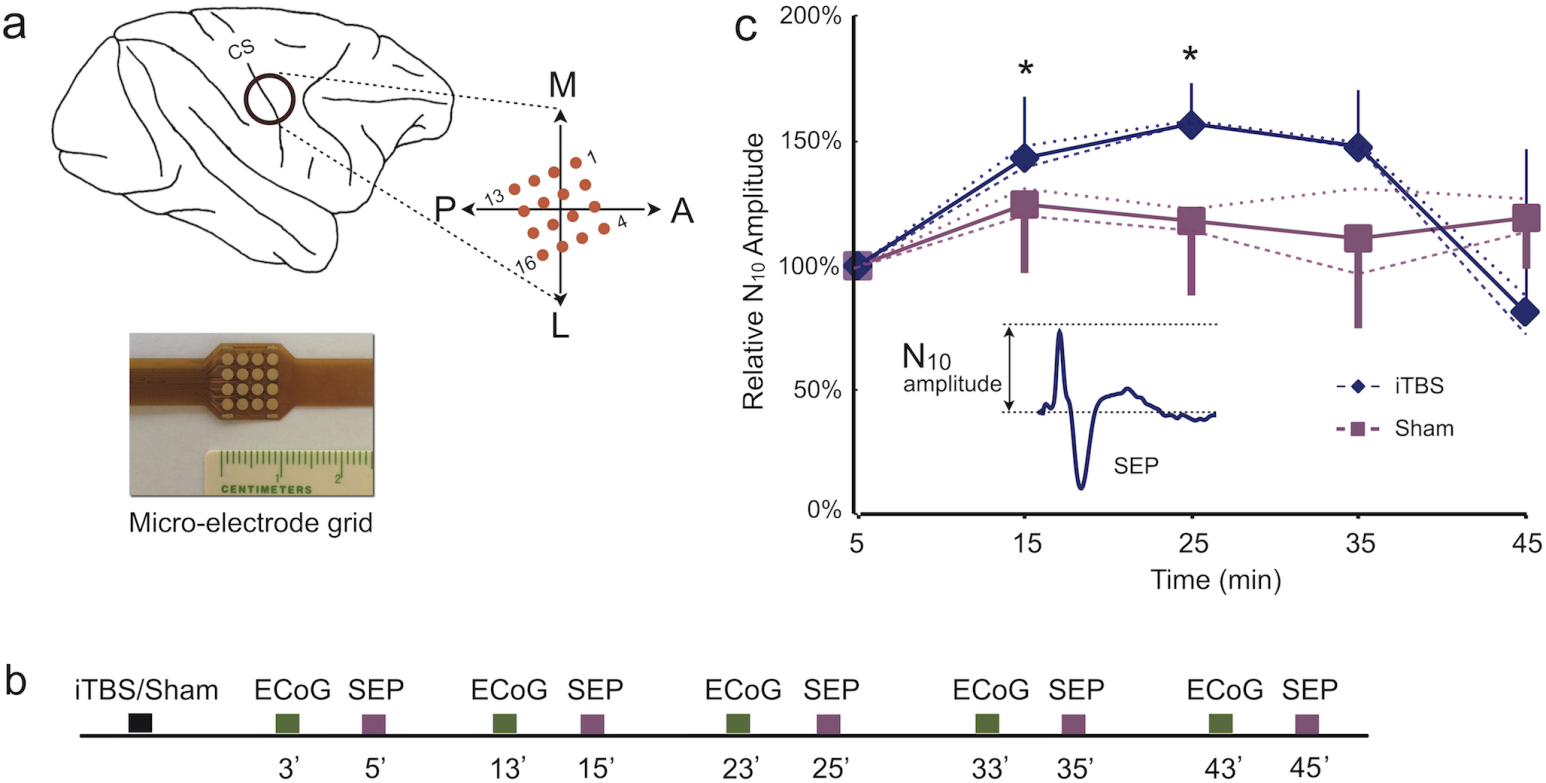
Effect of iTBS-induced LTP-like potentiation, a) schematic electrode distribution over the central sulcus (CS) and the actual epidural grid used during the experiments, b) experimental paradigm timeline, c) iTBS and sham stimulation effect on SEP amplitude. Amplitude is reported as ratios of the first time point (5th min) minute after real or sham iTBS stimulation. N_10_ indicates the signal’s negative deflection observed at a latency of about 10 ms in SEP responses (inset). Error bars are standard deviations. Dotted lines represent individual monkeys while continuous lines their average (*p<.01).

We performed the experiments in two Macaca mulatta monkeys. Under general anesthesia (isoflurane 1–3% to effect), we surgically mounted a permanent frontal headpost approximately over Fz and circular recording chamber (18 mm in diameter) for chronic neural activity recording over the right hemisphere granting access to the M_1_, the central sulcus and the S_1_ (Figure 1a). Surgical locations were measured stereotaxically and confirmed by visual inspection after dura opening at the end of the experiment in both animals.

All efforts were made to minimize suffering. Ten minutes prior each experimental session each monkey was sedated with a single shot of Metedomidine (0.02 mg/kg) which ensured approximately 45 minutes of sedation. During the experiments the monkeys were seated on a primate chair in a dimly lit room with their head, arms and legs immobilized. Animal care, housing and surgical procedures were in conformity with the European (Directive 2010/63/UE) and Italian (DD.LL. 116/92 and 26/14) laws on the use of non-human primates in scientific research and were approved (no. 132/2012-C) by the Italian Ministry of Health. The housing conditions and the experimental procedures were in accordance with the recommendations of the Weatherall report (for the use of non-human primates in research). Purpose-bread monkeys were pair-housed in primate cages (Tecniplast, Italy) in an illumination and temperature controlled ambient, and their health and welfare was monitored daily by the researchers and a designated veterinarian. We routinely introduced in the home cage environment, toys to promote their exploratory behaviour. Both monkeys have been used in other experimental protocols at the end of the experimental procedures here described.

### Transcranial Magnetic Stimulation Technique

**Figure 2:**
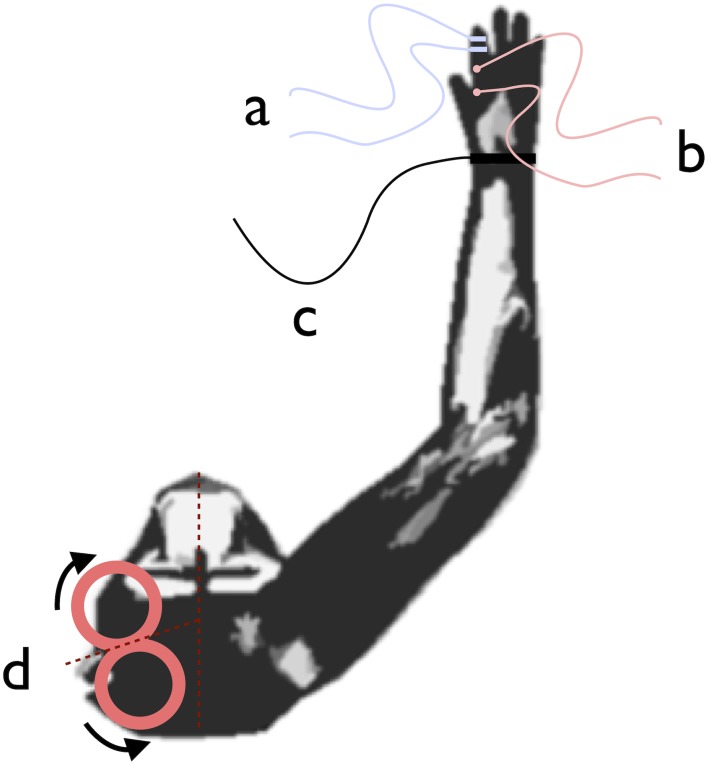
Schematic representation of peripheral nerve stimulation ring electrodes (a), surface electromyographic electrodes on the first dorsal interosseus muscle (b), ground electrode (c) and coil positioning (d) with arrows indicating the current flow.

We first applied test stimulations consisting of single pulse TMS delivered through a biphasic high-power magnetic stimulator (Magstim Rapid^2^, The Magstim Company Ltd, Whitland, South West Wales, UK) connected to a custom-made figure-of-eight coil with mean loop external and internal diameters of 7 cm and 2 cm, respectively and center-to-center loop distance of 6 cm, (Magstim Company Ltd). The magnetic stimulus had a biphasic waveform with a pulse width of ∼300 µs. During the first phase of the stimulus, the current in the centre of the coil flowed toward the handle. The coil was placed over the recording chamber on the optimum scalp position (hot spot) to elicit MEPs in the contralateral first dorsal interosseus muscle. In contrast to human studies, the optimal coil position over the monkey scalp in order to evoke MEPs was found to be tangentially to the scalp with the coil midline pointing away from the scalp midline at 72° inducing postero-anterior followed by antero-posterior (PA-AP) current in the brain (Figure 2). The distance between the coil surface and the brain was, because of the presence of the recording chamber, 17 mm ca. in both monkeys. However, this value is not different of the average coil-cortex distance reported in previous studies in humans [30].

Conditioning stimulation consisted of iTBS delivered over the recording chamber using the above experimental setup. iTBS was applied according to the technique described by Huang et al. (2005) [10] with the coil positioned as described earlier. iTBS consisted of a 2-sec train of magnetic stimulation with triplets of 50 Hz in a 5 Hz rhythm repeated every 10 sec for a total of 190 s (600 pulses) at 80% of the intensity necessary to evoke a 1 mV MEP [10]. It is important to stress that in the studied monkeys during sedation the intensity necessary to evoke a 1 mV MEP increased from an average of 33% to an average of 54% of the maximum stimulator output. Considering the difficulty in measuring active motor threshold in the awake monkey we chose to modify the original iTBS [10] protocol by setting the stimulator intensity slightly lower than the intensity necessary to evoke a 1 mV MEP in the awake monkey, but significantly lower from the intensity necessary to evoke a 1 mV MEP in the sedated monkey, at 27% of the maximum stimulator output. Sham iTBS was delivered with the coil placed orthogonally over the ‘hot spot’ defined earlier. Experimental sessions were performed with at least 7 days of interval between sessions.

### Electromiographic Recording

During test stimulation, in each animal we recorded surface electromyography (EMG) from the first dorsal interosseus muscle using a belly-tendon montage, with two gold plated Ag/AgCl recording electrodes and a ground electrode on the wrist (see Figure 2). The EMG signal was acquired with the notch filter on (45–55 Hz) and a bandpass filter implemented at the 20–1000****Hz band (Magstim Rapid^2^, The Magstim Company Ltd, Whitland, South West Wales, UK). The amplitude of MEPs recorded was measured peak to peak (mV) and then averaged. Test TMS pulses over the hot spot for the first dorsal interosseus muscle were applied in order to identify the intensity necessary to evoke a 1 mV MEP average out of 10 consecutive test TMS pulses.

### Electrocorticographic Recordings

We recorded continuous epidural ECoG from a custom-made multi-electrode grid, designed and implemented in collaboration with the ISS Lab (Rome) composed of sixteen 2 mm round gold plated electrodes with a 2.5 mm step on a Kapton substrate commonly referenced to the headpost, which ensured a Fz-like reference [31]. The impedance between the electrodes and the reference was kept below 5 kOhm. All recordings with impedance measurements above 5 kOhm were discarded, as high signal to noise ration was considered a key element of the study. Generally, impedance values remained bellow 1 kOhm. A grounding electrode was used on the ipsilateral auricular point. The signal from each electrode was amplified, digitised and optically transmitted to a digital signal processing unit were it was acquired at 6 kHz together with the stimulation trigger (Tucker-Davis Technologies, Alachua, FL).

### Median Nerve Electrical Stimulation and SEP Recordings

The median nerve was electrically stimulated using a pair of ring electrodes at the left index finger at the level of the collateral ligament between the 1st–2nd and 2nd–3rd phalanx with the cathode proximal and an inter-electrode distance of approximately 1 cm (Figure 2). Square wave pulses (width 0.15 ms; current 15 mA) were delivered with an S88 dual output square pulse stimulator paired to a PSIU6 photoelectric stimulus isolation unit (Grass Technologies, Astro-Med Inc, West Warwick, RI). A ground electrode was placed on the wrist of the hand that was stimulated to minimise the stimulus artifact. Each test experimental block consisted of 300 electric pulses delivered at 10 Hz.

For each conditioning session, the median nerve was stimulated every 10 minutes from 5 to 45 minutes after real or sham iTBS (Figure 1b). To extract evoked potentials from the signal, we selected epochs of 100 ms starting from the time of stimulus onset. The raw signal for each electrode was filtered using a bi-directional FIR bandpass filter (0.3–300****Hz, notch filter 45–55 Hz) and re-referenced to the average of all electrodes. For each electrode we removed the mean value and the linear trend of each epoch and then averaged the signal across epochs. Evoked components were identified as deflections of the signal amplitude that exceeded 2 standard deviations. The amplitude of the first negative deflection in the signal (N_10_; see Figure 1c, inset), used for statistical analysis, was measured using the electrode over S_1_ that presented the least variable signal during the first block and persisted during the session. Electrode location, over M_1_ or S_1_, was identified according to the phase inversion of the principal SEP components [32] and further confirmed by visual inspection (after surgically opening the dura at the end of the experiment) of the position of recording chamber vs the central sulcus.

### Time-Frequency Analysis

To extract iTBS-induced modulations we focused on five segments of the ECoG signal lasting 1 minute each, obtained every 10 minutes from 3 to 43 minutes after real or sham iTBS (Figure 1b). These segments where free of SEP and stimulation artifacts as SEPs where acquired with an offset of 2 minutes, from 5 to 45 minutes of the experimental session (see *Median Nerve Electrical Stimulation and SEP Recordings*). We calculated the modulations of the power spectral density (PSD) using Welch’s averaged modified periodogram method of spectral estimation. We applied the fast Fourier transform over chunks of 8192 points (1.34 sec), with Hanning windowing and 50% overlapping (0.67 sec) and averaged the modulus of the resulting time-frequency coefficient matrix, that is throughout the whole minute to get the mean power estimate (frequency resolution of 0.73 Hz) for each electrode [33]. In order to reduce the complexity of analysis and interpretation, we grouped the electrodes in M_1 (1–8)_, S_1 (9–16)_ and S_1_–M_1 (1–16)_ and calculated the arithmetical mean of the power in each group to get the average power for the underlying cortical areas.

We also calculated a coefficient of variation (C_V_) for the power of the signal, C_V,_ across selected electrodes, for every Fourier chunk considered.
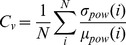



N (ca 90) stands for the number of chunks examined over the one minute period, σ is the variance of the values and µ the mean value across the electrodes encompassed in each group. C_V_ discloses the spatial constrains of the various oscillatory drives affected and studied. More autonomous activity of the underlying neural population should result in higher C_V_ values.

### Statistical Analysis

We first averaged the band power of the signals and the C_V_ for δ (1–4 Hz), θ (4–8 Hz), α (9–12 Hz), β (13–26 Hz) low γ (27–45 Hz) and high γ (55–90 Hz) bands.

In order to study the time evolution of iTBS aftereffects we expressed the SEP-N_10_ amplitude, the power spectral distribution and C_V_ values for each time point as ratios of the first time point values. We applied a repeated-measures ANOVA, with ‘Time’ (time point after real or sham iTBS) and ‘Stimulation’ (real iTBS, sham iTBS) as within-subject factors to compare the effect of stimulation on SEP-N_10_ amplitude. We also applied a repeated-measures ANOVA, with ‘Time’ (time point after real or sham iTBS), ‘Site’ (M_1_, S_1_, S_1_–M_1_) and ‘Stimulation’ (real iTBS, sham iTBS) as within-subject factors to compare the effect of stimulation on frequency band power and C_V_ modulations for each band (δ, θ, α, β, low γ, high γ). To disclose differences between subjects, we repeated the above statistics after adding the factor ‘Monkey’ (Tables S1–S3).

A Pearson rank correlation test was used to assess all possible correlations between TBS-induced changes in SEP-N_10_ amplitudes at all time points, significant power modulations and C_V_ across bands and sites.

All Post hoc comparisons were performed using Fisher’s Least Significant Difference test with the Bonferroni adjustment for multiple comparisons, as in all multiple comparisons here presented. In cases where Mauchly’s sphericity test was violated, Greenhouse-Geisser corrected P values where used. All data analysis procedures were implemented with custom routines using Matlab (Mathworks©, MA) and PASW© Statistics.

## Results

We performed a total of nine iTBS (five for monkey A and four for monkey I) and six sham experimental sessions (three for each monkey). Results were consistent across monkeys (Tables S1–S3). We observed that the SEP N_10_ component significantly increased in amplitude (Figure 1c) after real but not after sham iTBS [main effect for Time: F_1.566,20.353_ = 5.499, p = .017; main effect for Stimulation: F_1,13_ = 1.582, p = .231; Interaction Time*Stimulation: F_1.566,20.353_ = 6.103, p = .012] (Figure 1c). Post-hoc analysis (Bonferroni P = .01) revealed that the stimulation effect was significant at 15 (p = .005) and 25 (p = .3e-5) minutes after iTBS.

**Figure 3:**
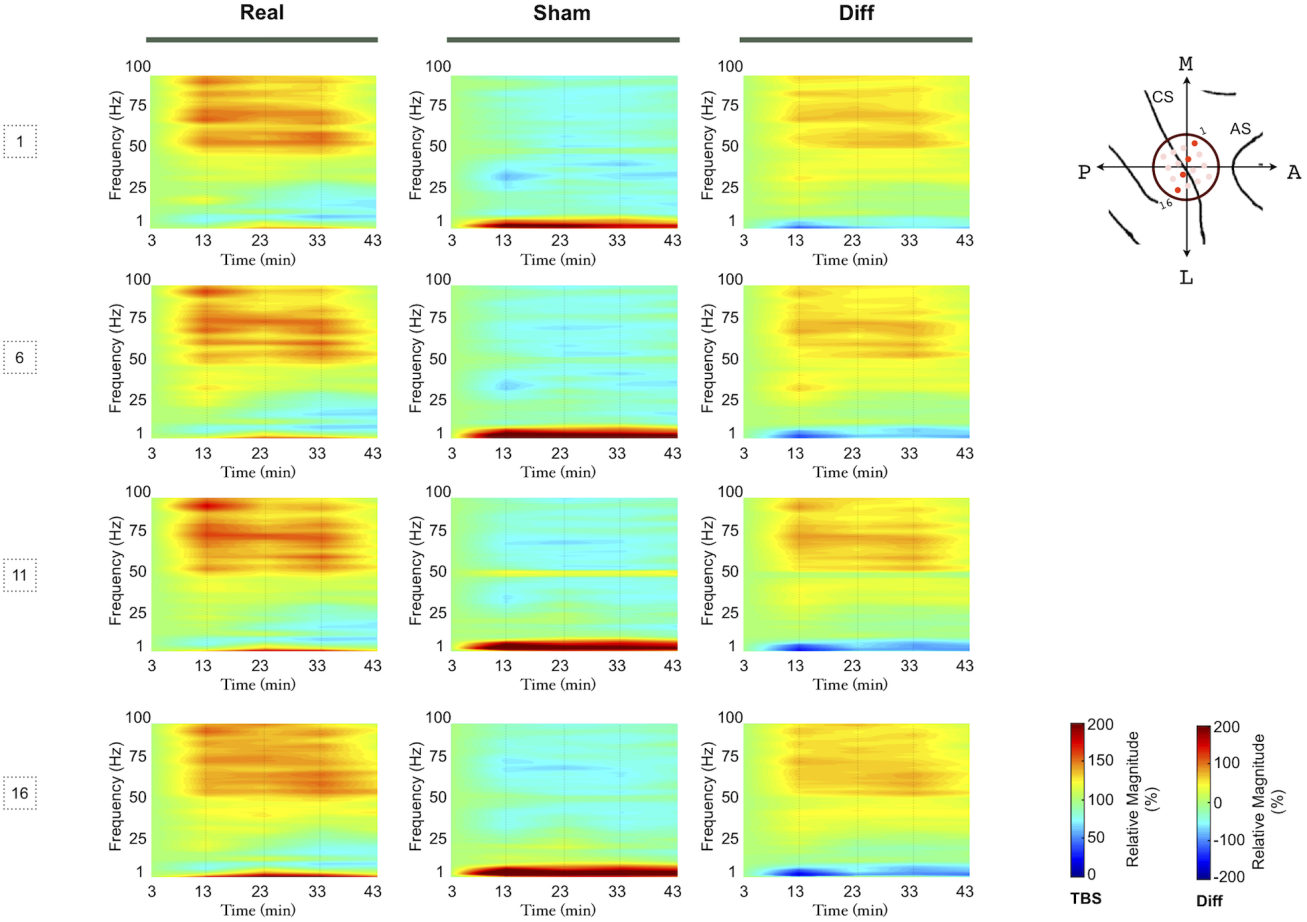
Power spectral density modulation of the signal from M_1_ (1 and 6) and S_1_ electrodes (11 and 16), as highlighted in the figure (CS: Central Sulcus, AS: Arcuate Sulcus), after iTBS and sham stimulation. Average values of nine sessions of real iTBS (Real) and six sessions of sham iTBS (Sham) are presented, as well as their spectral difference (Diff). Frequency power values for each time point are expressed as ratios of the first time point (3rd min) frequency power values. We interpolated the missing time in the figure by calculated isolines from the time points analysed.

**Figure 4:**
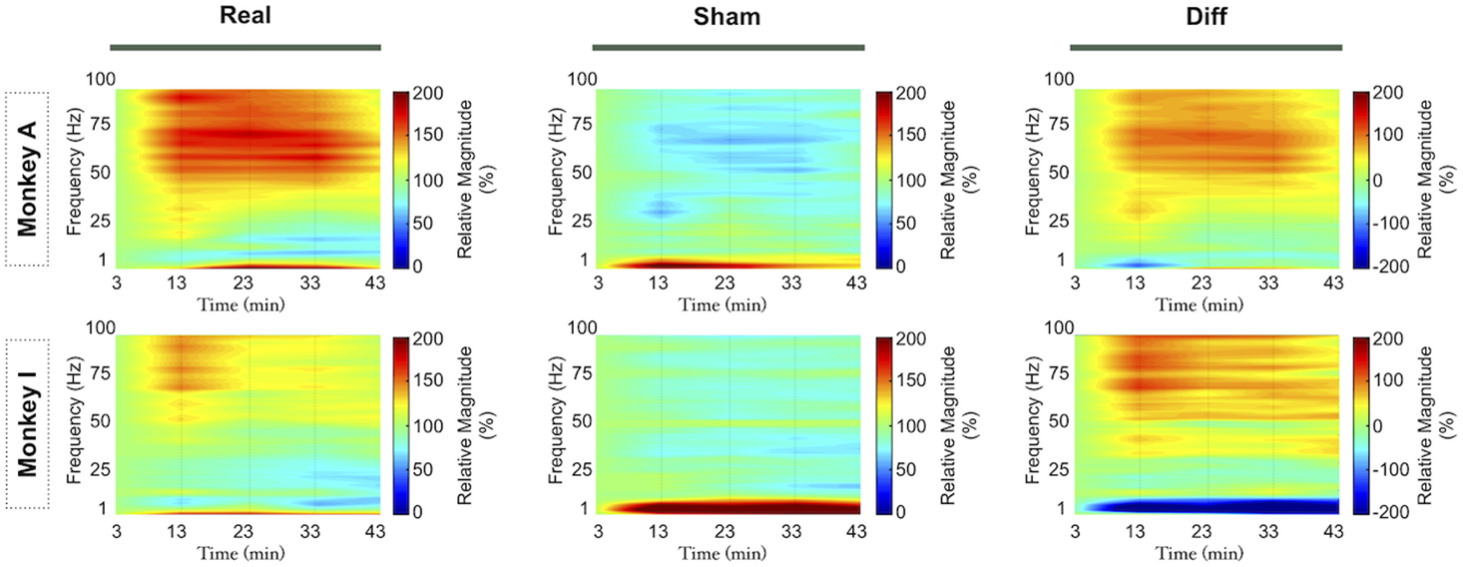
Power spectral density modulation for the signal obtained in the two animals. Real iTBS (Real), sham iTBS (Sham) and spectral difference (Diff) spectrograms for each monkey. Average values of all the electrodes are presented. Frequency power values for each time point are expressed as ratios of the first time point (3rd min) frequency power values. We interpolated the missing time in the figure by calculated isolines from the time points analysed.

**Figure 5:**
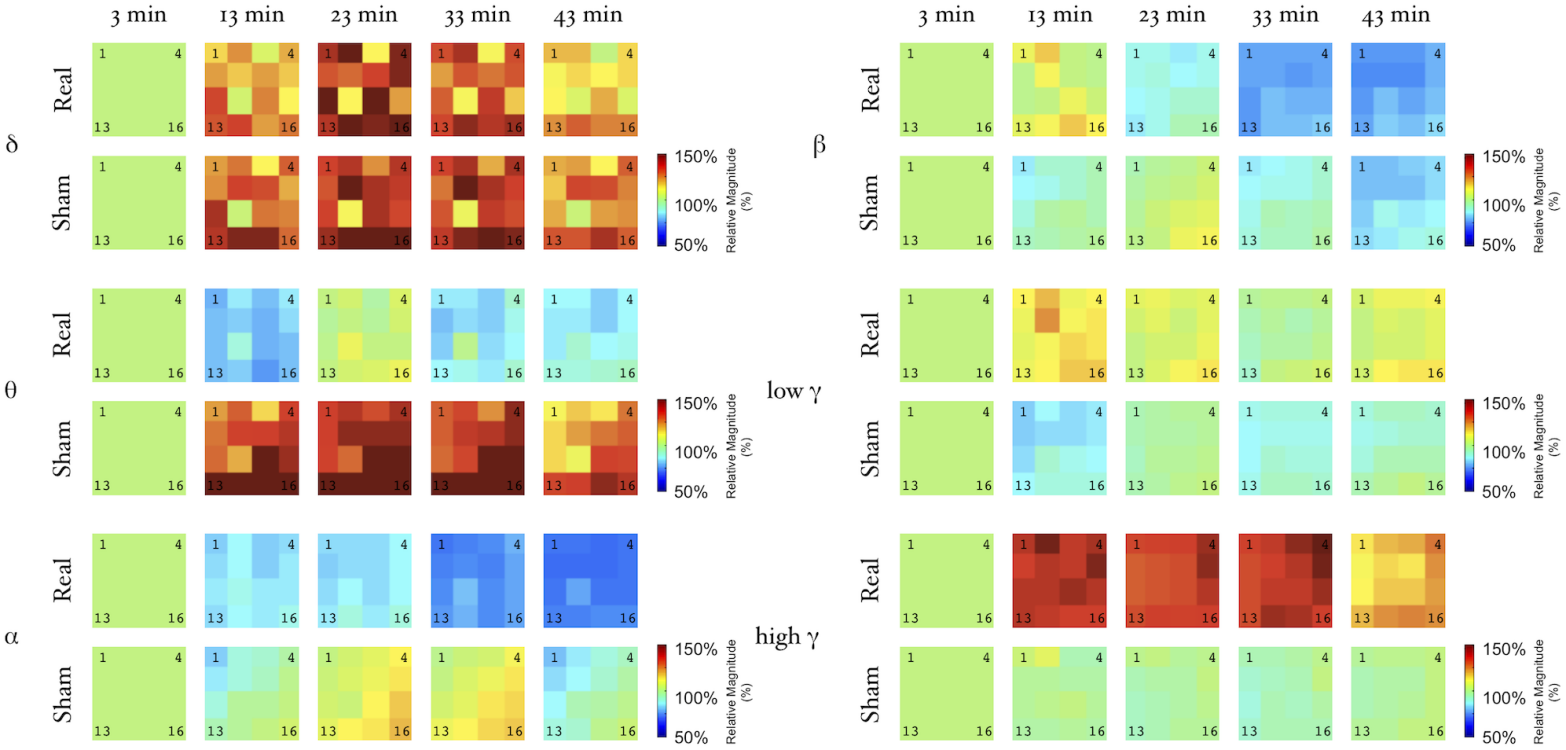
Topographic distribution of power modulation. We present in a grid representation, color-coded power modulation for each electrode of the 16, in every band considered and for every time point as refereed to the baseline period (3 min after iTBS stimulation).

We observed similar effects after iTBS over the different bands independently of the spatial location of the electrodes (Figure 3), animal (Figure 4) and electrodes (Figure 5). Repeated measures ANOVA disclosed a significant time trend in both real and sham iTBS sessions for the δ and β band power. We found significant Time-Stimulation interaction differences between real and sham iTBS sessions for the θ, α, low and high γ bands. Detailed results can be found in Table 1. Post-hoc analysis disclosed that real iTBS blocked the activity increase in the θ band, observed during the sham iTBS, at 13 (p = .0001), 23 (p = .005), 33 (p = .0007) and 43 (p = .0005) minutes and in the α band at 23 (p = .009), 33 (p = .001) and 43 (p = .00005) minutes. Conversely, iTBS promoted an activity increase in the low γ band at 13 (p = .000003), 23 (p = .003), 33 (p = .0006) and 43 (p = .0002) minutes after iTBS and high γ band at 13 (p = .000001), 23 (p = .000003) 33 (p = .00005) and 43 (p = .00007) minutes after iTBS. These results were Site independent (see Table 1), thus iTBS induces aftereffects on the signal power of both S_1_ and M_1_ neural oscillatory activity.

**Table 1:** RM-ANOVA results for band power modulation in Time (3 min, 13 min, 23 min, 33 min, 43 min) Stimulation (iTBS, Sham) and Site (M_1_, S_1_, M_1_–S_1_). Bonferroni corrected P values<.008 (values in bold) were considered significant.

	*δ (1–4 Hz)*	*θ (5–7 Hz)*	*α (8–12 Hz)*	*β (13–26 Hz)*	*low γ* *(27–45 Hz)*	*high γ* *(55–90 Hz)*
*Time*	**F(2.648, 103.2) = 12.141,** **p = 2e-6**	F(2.591, 101.05) = 4.031,p = .013	F(2.583, 100.72) = 3.297p = .03	**F(2.126, 82.9) = 11.157,** **p = 5e-8**	F(2.664, 103.9) = 4.422,p = .008	F(2.661, 103.78) = 1.354,p = .252
*Time* *Stimulation	F(2.648, 103.2) = 1.228, p = .3	**F(2.591, 101.05) = 8.653,** **p = 8e-6**	**F(2.583, 100.72) = 5.432,** **p = .003**	F(2.126, 82.9) = 1.905,p = .153	**F(2.664, 103.9) = 10.036,** **p = 1e-5**	**F(2.661, 103.78) = 12.99,** **p = 8e-7**
Time*Site	F(5.296, 103.2) = .015, p = 1	F(5.182, 101.05) = .026,p = 1	F(5.165, 100.72) = .065,p = .998	F(4.251, 82.9) = .035,p = .998	F(5.328, 103.9) = .032,p = 1	F(5.322, 103.78) = .034,p = 1
Time*Site *Stimulation	F(5.296, 103.2) = .11, p = 1	F(6.295, 101.05) = .032,p = 1	F(5.165, 100.72) = .065,p = .997	F(4.251, 82.9) = .023,p = .999	F(5.328, 103.9) = .009,p = 1	F(5.322, 103.78) = .014,p = 1
*Stimulation*	F(1,39) = .979.,p = .33	**F(1,39) = 14.536,** **p = 4e-4**	**F(1,39) = 0.2,** **p = .004**	F(1,39) = .164,p = .688	**F(1,39) = 20.433,** **p = 5e-5**	**F(1,39) = 30.693,** **p = 2e-6**
*Site*	F(2,39) = .003,p = .997	F(2,39) = .044,p = .95	F(2,39) = .15,p = .861	F(2,39) = .107,p = .898	F(2,39) = .06,p = .942	F(2,39) = .019,p = .981
*Stimulation* *Site	F(2,39) = .02,p = .98	F(2,39) = .051,p = .95	F(2,39) = .146,p = .864	F(2,39) = .057,p = .945	F(2,39) = 4e-4,p = 1	F(2,39) = .016,p = .984

**Figure 6:**
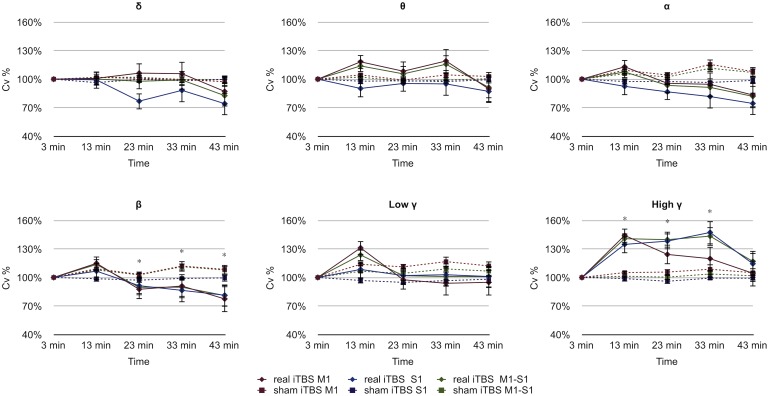
Modulation of the C_V_ for the bands considered. Real iTBS aftereffects are marked by continuos lines while dashed lines indicate sham iTBS aftereffects. Note the raise of the high γ band C_V_ in M_1_, S_1_ and M_1_–S_1_ after real iTBS and the small but significant fall of the β band (*p<.01).

Repeated measures ANOVA with ‘Time’ (time point after real or sham iTBS), ‘Site’ (M_1_, S_1_, S_1_–M_1_) and ‘Stimulation’ (real iTBS, sham iTBS), as within-subject factors for each band (δ, θ, α, β, low γ, high γ) disclosed significant C_V_ modulation after iTBS for the β band for Time-Stimulation interaction and for the high γ band for Time, Stimulation and Time-Stimulation interaction (details in Table 2). Post-hoc analysis disclosed that C_V_ for the β band decreased significantly at 23 minutes (p = .009), 33 minutes (p = .001) and 43 minutes (p = .003) after real stimulation, as compared to the sham stimulation and C_V_ for the high γ band increased significantly at 13 minutes (p = .0004), 23 minutes (p = .001) and 33 minutes (p = .001) after real stimulation, as compared to the sham stimulation (Figure 6).

**Table 2:** RM-ANOVA results for the coefficient of variability (C_V_) modulation in Time (3 min, 13 min, 23 min, 33 min, 43 min) Stimulation (iTBS, Sham) and Site (M_1_, S_1_, M_1_–S_1_).

	*δ* *(1–4 Hz)*	*θ* *(5–7 Hz)*	*α* *(8–12 Hz)*	*β (13–26 Hz)*	*low γ* *(27–45 Hz)*	*high γ* *(55–90 Hz)*
*Time*	F(3.215, 125.395) = 2.153, p = .092	F(3.028, 118.111) = 2.058, p = .109	F(2.610, 101.8) = 2.506,p = .071	F(2.478, 96.623) = 4.465,p = .009	F(2.101, 81.928) = 2.654,p = .074	**F(3.221, 125.62) = 5.978,** **p = .001**
*Time* *Stimulation	F(3.215, 125.395) = 1.814, p = .144	F(3.028, 118.111) = 2.135, p = .099	F(2.610, 101.8) = 4.044,p = .013	**F(2.478, 96.623) = 5.035,** **p = .005**	F(2.101, 81.928) = 2.080,p = .129	**F(3.221, 125.62) = 4.999,** **p = .002**
Time*Site	F(6.430, 125.395) = .377, p = .902	F(6.057, 118.111) = .688, p = .661	F(5.220, 101.8) = .532,p = .759	F(4.955, 96.623) = .227,p = .949	F(4.201, 81.928) = .457,p = .776	F(6.442, 125.62) = .256,p = .963
Time*Site *Stimulation	F(6.430, 125.395) = .479, p = .834	F(6.057, 118.111) = .322, p = .926	F(5.220, 101.8) = .090, p = .995	F(4.955, 96.623) = .084,p = .994	F(4.201, 81.928) = .435,p = .792	F(6.442, 125.62) = .450,p = .855
*Stimulation*	F(1, 39) = 1.109,p = .299	F(1, 39) = .172,p = .680	F(1, 39) = 6.801,p = .013	F(1, 39) = 6.880,p = .012	F(1, 39) = .012,.p = .914	**F(1, 39) = 12.691,** **p = .001**
*Site*	F(2, 39) = .649,p = .528	F(2, 39) = 1.431,p = .251	F(2, 39) = 2.166,p = .128	F(2, 39) = .624,p = .541	F(2, 39) = .828,p = .444	F(2, 39) = .082,p = .922
*Stimulation* *Site	F(2, 39) = .416,p = .662	F(2, 39) = .608,p = .549	F(2, 39) = .004,p = .996	F(2, 39) = .295,p = .746	F(2, 39) = .634,p = .536	F(2, 39) = .526,p = .595

Bonferroni corrected P values<.008 (values in bold) were considered significant.

Pearson correlation test showed that the C_V_ decrease of the β band was correlated to the β band power decrease in the S_1_ (r = .979, p = .004), while the C_V_ increase of the high γ band was correlated to the transient increase of the high γ band power in S_1_ (r = .974, p = .005) and M_1_–S_1_ (r = .993, p = .001). No significant correlation was found with SEP-N_10_ amplitude and band power or C_V_ (Table S4).

## Discussion

In our study, we explored the iTBS induced neural activity modulation obtained from a custom miniaturised ECoG grid, surgically placed over the sensorimotor region in two sedated monkeys. Our results show that LTP-like aftereffects induced by iTBS modulate the spectral imprint of rhythmic activity both locally and in the interconnected network. iTBS induced changes of local neural interactions, attested by the significant modulation of the power spectrum, and distributed changes attested by the C_V_. Regarding the iTBS-induced aftereffects on N_10_, we confirm the previously reported observations made on humans [22], [23].

Several authors reported that TMS pulses over the scalp modulate cortical activity. In a co-registration EEG-TMS study Paus and colleagues (2001) [17], reported that single pulse TMS applied over M_1_ induced highly synchronous oscillatory activity in the β band that lasted several hundred milliseconds, a finding confirmed by Fuggetta and colleagues (2005) [18] who further showed that single TMS pulses produced neural synchronisation both in α and β range which increased linearly with the TMS intensity within 500 ms. In extracellular recordings, Allen and colleagues [19] observed that immediately after a TMS pulse, oscillatory activity in the γ band tends to increase while lower bands tend to decrease in power. These oscillatory modulations are probably linked to the resetting of the ongoing oscillatory activity and could be considered part of a wider, recently conceived neurophysiological measurements applied to study cortical connectivity and excitability, termed transcranial evoked potentials (TEPs, [36]). However, long lasting modulations of naturally occurring cortical rhythms may require long-term local cortical activity modulation as well as functional reorganization of the neural connections involved. Thus further study of continuous brain rhythmic activity modulation induced by TMS is opportune to clarify the long term aftereffects attributed to LTP-like plasticity.

To our knowledge there are only two published studies that explored the influence of TBS in naturally occurring rhythms, one by Saglam and colleagues (2008) and one by McAllister and colleagues (2012) [11], [12]. Both studies, present EEG activity from healthy humans after having applied the continuous TBS paradigm and conclude that though TBS seems to influence rhythmic activity, power modulation never reaches statistical significance. Contrary to trial-averaged event-related studies, continuous EEG recordings do not contain a persistent and recurrent signal that blends with a noisy background and can be brought to light by averaging several trials, but a subtle, more variant signal that is virtually indistinguishable from the noisy background [24], [25]. Thus, the lower signal to noise ratio and the spatial blurring of EEG within and between subjects could be responsible for the negative results.

An important difference with respect to previous studies is that in our study subjects were examined in a sedated state. In a sedated state, pulse propagation is compromised, cortical response is amplified, and rhythmic activity is shifted towards slower oscillations [37]. Metedomedine, the administered sedative, is an α_2_-agonist that induces a sleep-like state, promoting reliable sedation and anxiolysis [38], [39], [40]. These effects are mediated by α_2_ receptors located primarily in locus coeruleus neurons on the pons and lower brainstem [38], [39], [40]. Thus, Metedomedine does not disrupt the local cortical network, nor it interferes directly with NMDA or GABA transmission, but it probably provides a state of lesser inputs to the S_1_ and M_1_ [38], [39], which could make the neural populations of these cortical areas more susceptible to external influence by means of TBS. In fact, noradrenergic activity in the thalamus has a blocking effect on the relay cells through α-2 adrenoceptors located on the thalamocortical neurons [38]. Interestingly, the muscle relaxation effect that accompanies sedation is due to inhibition of α_2_-receptors at the interneuron level of the spinal cord, which is in line with the significant difference in the intensity necessary to evoke a 1 mV MEP that we found between sedated and awake states [40]. Additionally, significant modulations in time observed for δ and β bands can be related to different stages of sedation that evolves from deep to superficial at the end of the experimental session, when Metedomedine washes off.

A less critical difference between the experimental paradigm here described and other similar paradigms [22], [23] is the assumption of a baseline immediately after instead of before the iTBS conditioning. We adopted the former approach for two main reasons. Firstly, we decided not to stimulate through the multi-electrode grid in order to avoid magnetic line deformation and exposure of the animal to possible risks due to heating of the metal elements. Secondly, though multi-electrode grid positioning was controlled by anatomical landmarks, manipulation of the multi-electrode grid during testing sessions occasionally resulted in failure to maintain precise adherence and position, that we considered to be crucial in order to compare the different time points during each session. Thus, the multi-electrode grid was applied immediately after each iTBS conditioning protocol and removed daily at the end of the experimental session.

Classically, iTBS aftereffects grow from 3–15 min and maximum aftereffects are observed at 15–25 min interval after iTBS conditioning [10], [22], [23]. Additionally, iTBS aftereffects tend to recede at 35–45 min after iTBS conditioning. Hence, in our experimental paradigm we included most of the expected iTBS-induced modulated neural activity.

A crucial parameter of the iTBS protocol is the TMS stimulation intensity. A single high-intensity TMS pulse induces massive cortical activation lasting seconds, distributed across layers, with interneurons showing the shortest latency to respond, followed by axonal activation of thalamo-cortical and cortico-cortical afferent fibers [41], [42]. In M_1_, the resulting indirect activation of pyramidal cells, often generated by long polysynaptic networks or recurrent synaptic networks, induces the propagation of a cortico-spinal action potential and an observable MEP [41]. It has been suggested that iTBS, delivered at comparatively low intensities, unable to evoke a MEP, targets mainly interneurons, in contrast to high-intensity TMS pulses [10].

Interneuronal involvement in the iTBS aftereffects is also supported by the modulation of the power spectral density in our data. Interneuron activity is closely related to both θ and γ band activity [1], [43], [44]. In vivo, in vitro and simulation data portray the interneurons as the principal neural population involved in γ band oscillatory activity modulation [1], [44], [45], [46]. In the neocortex, γ band activity has been observed to originate form superficial layers 2/3 that mainly include interneurons [47], while recently Ahmed and Mehta (2012) [48] proposed that γ band modulations are correlated with changes in the firing rate of individual interneurons also in the hippocampus. Thus, the γ band power modulation we observed in sensorimotor cortex further supports the hypothesis that iTBS increases cortical excitability by affecting interneurons.

We found modulations in both high and low frequency band power in both monkeys (Figure 4). Field potentials from epidural electrodes highlight the common action of neurons because of neural topographical arrangement combined with the dynamic coordination of neurons and depend on the underlying functional network [34]. As higher frequency oscillatory activity are thought to reflect the coordination of smaller ensembles of cortical neurons [51], [52], the rise in γ power is consistent with the activation of local cortical loops [35], also supported by the global rise of the C_V_. Lower oscillation frequencies on the other hand reflect long-range coupling [2], [4], [50], so that diminished θ and α power may indicate the disruption of a large-scale distributed network, possibly involving thalamo-cortical couplings. In summary, the results we report here suggest that after iTBS conditioning, local sets of neurons are brought together into coherent ensembles, while remote connections are inhibited, thereby establishing a long-lasting reorganisation [35], [52].

To measure whether the ECoG activity observed simultaneously in selected electrodes was clustered or dispersed after real iTBS, with respect to sham iTBS, for each considered electrode group (S_1_, M_1_, S_1_–M_1_) we computed the C_V_ measure (see Methods). For each time segment, the more inhomogeneous the electrical activity was, the higher was the C_V_. After real iTBS high γ band C_V_ increased transiently and fell back towards the end of the experimental session, while after sham iTBS high γ band C_V_ remained globally uninfluenced (Figure 6). Conversely, iTBS aftereffects on β band C_V_ decreased after real iTBS and never completely recovered during the experimental session, while after sham iTBS, again, β band C_V_ remained uninfluenced globally. Thus, regional activity in the β band was more homogeneous while the activity in the γ band was increased and more irregular. One explanation may come from the local origin of both bands. In the neocortex, γ band oscillations are prominent in layers 2/3, which contains cells of similar properties that mainly project horizontally, while beta-band oscillations seem to originate from layers 5/6 [47], [49]. It has been proposed that a regulating network between these two neural populations exists [47], [49]. Neuronal populations situated in layers 2/3 generate synchronous oscillations in the γ band that propagate information to layers 5/6, which generate oscillations in the β band, under some circumstances [47], [49]. The role of the layers 2/3 neural populations in this network is feed-forward, while layers 5/6 populations have a feed-back role [47], [49]. We believe that rise in magnitude and irregularity of the γ band is consistent with a feed-forward mechanism that evolves in local synchronisation, while more regular β band activity reflects a broader feed-back process. It should also be noted that while high γ band C_V_ modulations have an early onset, β band C_V_ modulations arise only after γ band modulations reach significant levels. While more experiments are needed to provide an accurate accounting of our results, our data indicate that iTBS also modulates local cortical activity distributions.

Our study had some limitations. Although iTBS induces mainly focal LTP-like aftereffects and we used a small focal figure-of-eight coil, we found significant modulation in most of the electrodes, spanning from M_1_ to S_1_, as the topographical distribution of induced currents comprises the whole recording chamber. Moreover, a distant epicortical or deep brain recording would be of extreme usefulness in interpreting our data. Finally, it is difficult to generalize the outcome of our study in non-human primates to humans.

In conclusion, we found that iTBS modulated the spectral imprint of the area studied. We propose that spectral modulation is achieved by transiently reorganising the synaptic strength of interneurons promoting local cortical high-frequency oscillation and inducing a transient “deafferentation” of the targeted neural population. The down-modulation in the θ band (quite homogeneous among electrodes) suggests that this reorganisation could disrupt large-scale coordination. Our findings are in line with the accepted view that LTP phenomena underlie iTBS aftereffects. The similarity of our results to the θ and γ oscillatory activity modulation found in the hippocampus circuitry during LTP protocols [2] may point to a common plasticity backbone in both the neocortex and the hippocampus. Recently Siebner and Ziemann (2010) [53] cautiously drew a relationship between the frequency of naturally occurring neural oscillatory activity and those induced by external stimuli, speculating on the role the latter have in effectively boosting cortical oscillations. Further studies will establish the exact mechanism through which iTBS induces plastic after-effects. The lack of invasive studies examining the mechanism underlying TMS-induced LTP-like plasticity is remarkable. Considering that TMS techniques are routinely applied on humans, knowledge gaps should be filled for safety, methodological and neurophysiological reasons.

## Supporting Information

Table S1 **RM-ANOVA results for SEP N_10_ modulation in Time (3 min, 13 min, 23 min, 33 min, 43 min), Stimulation (iTBS, Sham) and Monkey (Monkey I vs Monkey A).**
(DOC)

Table S2 **RM-ANOVA results for band power modulation in Time (3 min, 13 min, 23 min, 33 min, 43 min), Stimulation (iTBS, Sham), Site (M1, S1, M1–S1) and Monkey (Monkey I vs Monkey A).**
(DOC)

Table S3 **RM-ANOVA results for coefficient of variation (C_v_) modulation in Time (3 min, 13 min, 23 min, 33 min, 43 min), Stimulation (iTBS, Sham), Site (M_1_, S_1_, M_1_–S_1_) and Monkey (Monkey I vs Monkey A).**
(DOC)

Table S4 **Correlation coefficient between SEP N_10_ amplitude, band power and coefficient of variation Cv for each Site (M_1_, S_1_, M_1_–S_1_).**
(DOC)
