# Supplementary material for: iTBS-Induced LTP-Like Plasticity Parallels Oscillatory Activity Changes in the Primary Sensory and Motor Areas of Macaque Monkeys
Source: PLoS One. 2014 Nov 10;9(11):e112504. doi: 10.1371/journal.pone.0112504 (PMC4226540; doi:10.1371/journal.pone.0112504)
Supplement: Table S1 — RM-ANOVA results for SEP N10 modulation in Time (3 min, 13 min, 23 min, 33 min, 43 min), Stimulation (iTBS, Sham) and Monkey (Monkey I vs Monkey A). (DOC) [file pone.0112504.s001.doc]

Table S1. RM-ANOVA results for SEP N_10_ modulation in Time (3min, 13min, 23min, 33min, 43min), Stimulation (iTBS, Sham) and Monkey (Monkey I vs Monkey A). P values < .05 (values in bold) were considered significant.

|  | SEP N_10_ |
| --- | --- |
| *Time* | **F(1.366, 14.028)=5.131, p=.03** |
| *Time*Mk* | F(1.366, 14.028)=.497, p=.549 |
| *Time*Stimul* | **F(1.366, 14.028)=5.682, p=.023** |
| *Time*Mk*Stimul* | F(1.366, 14.028)=.703, p=.458 |
|  |  |
| *Mk* | F(1, 11)=.229, p=.642 |
| *Stimul* | F(1, 11)=1.417, p=.259 |
| *Stimul*Mk* | F(1, 11)=.403, p=.539 |
