# Supplementary material for: iTBS-Induced LTP-Like Plasticity Parallels Oscillatory Activity Changes in the Primary Sensory and Motor Areas of Macaque Monkeys
Source: PLoS One. 2014 Nov 10;9(11):e112504. doi: 10.1371/journal.pone.0112504 (PMC4226540; doi:10.1371/journal.pone.0112504)
Supplement: Table S2 — RM-ANOVA results for band power modulation in Time (3 min, 13 min, 23 min, 33 min, 43 min), Stimulation (iTBS, Sham), Site (M1, S1, M1–S1) and Monkey (Monkey I vs Monkey A). (DOC) [file pone.0112504.s002.doc]

Table S2. RM-ANOVA results for band power modulation in Time (3min, 13min, 23min, 33min, 43min), Stimulation (Stimul) (iTBS, Sham), Site (M1, S1, M1-S1) and Monkey (Mk) (Monkey I vs Monkey A). Bonferroni corrected P values < .008 (values in bold) were considered significant.

|  | δ (1-4 Hz) | θ (5-7 Hz) | α (8-12 Hz) | β (13-26 Hz) | low γ (27-45 Hz) | high γ (55-90 Hz) |
| --- | --- | --- | --- | --- | --- | --- |
| *Time* | **F(2.522, 83.234)=11.15, p=1e-5** | F(2.663, 87.898)=3.83, p=.016 | F(2.589, 85,435)=2.958 p=.044 | **F(2.078, 66.287)=9.997, p=1e-4** | F(2.679, 81.909)=3.887, p=.015 | F(2.492, 91.71)=1.447, p=.239 |
| *Time*Stimul* | F(2.522, 83.234)=1.216, p=.307 | **F(2.663, 87.898)=8.225, p=1e-4** | **F(2.589, 85,435)=4.62 p=.007** | F(2.078, 66.287)=1.659, p=.198 | **F(2.679, 81.909)=8.700, p=8e-6** | **F(2.492, 91.71)=11.987, p=6e-6** |
| *Time*Site* | F(5.044, 83.234)=.017, p=1 | F(5.327, 87.898)=.024, p=1 | F(5.178, 85.435)=.057 p=.998 | F(4.017, 66.287)=.033, p=.998 | F(5.357, 81.909)=.026, p=1 | F(4.985, 91.71)=.050, p=.998 |
| *Time*Site*Stimul* | F(5.044, 83.234)=.01, p=1 | F(5.327, 87.898)=.03, p=1 | F(5.178, 85.435)=.058 p=.998 | F(4.017, 66.287)=.022, p=.999 | F(5.357, 81.909)=.008, p=1 | F(4.985, 91.71)=.040, p=.999 |
| *Time*Mk* | F(2.522, 83.234)=2.173, p=.108 | F(2.663, 87.898)=1.598, p=.2 | F(2.589, 85,435)=.396 p=.727 | F(2.078, 66.287)=1.063, p=.353 | F(2.679, 81.909)=1.254, p=.295 | F(2.492, 91.71)=.409, p=.710 |
| *Time*Stimul*Mk* | F(2.522, 83.234)=1.727, p=.176 | F(2.663, 87.898)=2.335, p=.086 | F(2.589, 85,435)=1.244 p=.298 | F(2.078, 66.287)=1.076, p=.348 | F(2.679, 81.909)=.371, p=.751 | F(2.492, 91.71)=.665, p=.549 |
| *Time*Site*Mk* | F(5.044, 83.234)=.038, p=.999 | F(5.327, 87.898)=.078, p=.997 | F(5.178, 85.435)=.021 p=1 | F(4.156, 66.287)=.003, p=1 | F(5.357, 81.909)=.018, p=1 | F(4.985, 91.71)=.008, p=1 |
| *Time*Site*Stimul*Mk* | F(5.044, 83.234)=.041, p=.999 | F(5.327, 87.898)=.074, p=.997 | F(5.178, 85.435)=.029 p=1 | F(4.156, 66.287)=.016, p=1 | F(5.357, 81.909)=.008, p=1 | F(4.985, 91.71)=.302, p=.910 |
|  |  | | | | | |
| *Stimul* | F(1, 33)=1.067, p=.309 | **F(1, 33)=14.144, p=.001** | **F(1, 33)=8.046, p=.0008** | F(1, 33)=.041, p=.841 | **F(1, 33)=17.095, p=2e-5** | **F(1, 33)=28.289, p=7e-6** |
| *Site* | F(2, 33)=.004, p=.996 | F(2, 33)=.043, p=.959 | F(2, 33)=.134, p=.875 | F(2, 33)=.118, p=.889 | F(2, 33)=.047, p=.954 | F(2, 33)=.023, p=.977 |
| *Mk* | F(1, 33)=4.699, p=.063 | F(1, 33)=2.908, p=.098 | F(1, 33)=.477, p=.495 | F(1, 33)=2.895, p=.098 | F(1, 33)=.058, p=.811 | F(1, 33)=.252, p=.619 |
| *Stimul*Site* | F(2, 33)=.017, p=.983 | F(2, 33)=.05, p=.952 | F(2, 33)=.132, p=.877 | F(2, 33)=.064, p=.938 | F(2, 33)=1e-4, p=1 | F(2, 33)=.024, p=.976 |
| *Stimul*Mk* | F(1, 33)=.436, p=.514 | F(1, 33)=2.641, p=.114 | F(1, 33)=1.982, p=.169 | F(1, 33)=.543, p=.466 | F(1, 33)=.018, p=.895 | F(1, 33)=1.218, p=.278 |
| *Site*Mk* | F(2, 33)=.048, p=.954 | F(2, 33)=0.118, p=.889 | F(2, 33)=.027, p=.973 | F(2, 33)=2e-4, p=1 | F(2, 33)=.012, p=.988 | F(2, 33)=.029, p=.972 |
| *Stimul*Site*Mk* | F(2, 33)=.142, p=.869 | F(2, 33)=.12 p=.997 | F(2, 33)=.024, p=.977 | F(2, 33)=.003, p=.997 | F(2, 33)=.002, p=.998 | F(2, 33)=.109, p=.897 |
