# Supplementary material for: iTBS-Induced LTP-Like Plasticity Parallels Oscillatory Activity Changes in the Primary Sensory and Motor Areas of Macaque Monkeys
Source: PLoS One. 2014 Nov 10;9(11):e112504. doi: 10.1371/journal.pone.0112504 (PMC4226540; doi:10.1371/journal.pone.0112504)
Supplement: Table S3 — RM-ANOVA results for coefficient of variation (Cv) modulation in Time (3 min, 13 min, 23 min, 33 min, 43 min), Stimulation (iTBS, Sham), Site (M1, S1, M1–S1) and Monkey (Monkey I vs Monkey A). (DOC) [file pone.0112504.s003.doc]

Table S3. RM-ANOVA results for coefficient of variation (C_v_) modulation in Time (3min, 13min, 23min, 33min, 43min), Stimulation (Stimul) (iTBS, Sham), Site (M_1_, S_1_, M_1_-S_1_) and Monkey (Mk) (Monkey I vs Monkey A). Bonferroni corrected P values < .008 (values in bold) were considered significant.

|  | δ (1-4 Hz) | θ (5-7 Hz) | α (8-12 Hz) | β (13-26 Hz) | low γ (27-45 Hz) | high γ (55-90 Hz) |
| --- | --- | --- | --- | --- | --- | --- |
| *Time* | F(3.09, 101.965)=2.181, p=.093 | F(3.097, 106.99)=2.095, p=.104 | F(2.533, 83.594)=1.538 p=.216 | F(2.273, 75.004)=4.059, p=.017 | F(2.073, 68.393)=2.306, p=.105 | **F(3.096, 102.153)=4.942, p=.003** |
| *Time*Stimul* | F(3.09, 101.965)=1.855, p=.140 | F(3.097, 106.99)=2.176, p=.093 | F(2.533, 83.594)=3.012 p=.043 | **F(2.273, 75.004)=4.711, p=.008** | F(2.073, 68.393)=1.790, p=.174 | **F(3.096, 102.153)=4.240, p=.007** |
| *Time*Site* | F(6.18, 101.965)=.359, p=.907 | F(6.193, 106.99)=.626, p=.714 | F(5.066, 83.594)=.635 p=.675 | F(4.546, 75.004)=.198, p=.953 | F(4.145, 68.393)=.347, p=.852 | F(6.191, 102.153)=.220, p=.972 |
| *Time*Site*Stimul* | F(6.18, 101.965)=.447, p=.851 | F(6.193, 106.99)=.296, p=.941 | F(5.066, 83.594)=.150 p=.980 | F(4.546, 75.004)=1.938, p=.145 | F(4.145, 68.393)=.338, p=.858 | F(6.191, 102.153)=.382, p=.893 |
| *Time*Mk* | F(3.09, 101.965)=.798, p=.501 | F(3.097, 106.99)=.558, p=.649 | F(2.533, 83.594)=1.118 p=.341 | F(2.273, 75.004)=1.245, p=.296 | F(2.073, 68.393)=.246, p=.790 | F(3.096, 102.153)=.791, p=.505 |
| *Time*Stimul*Mk* | F(3.09, 101.965)=1.27, p=.289 | F(3.097, 106.99)=1.525, p=.211 | F(2.533, 83.594)=1.105 p=.346 | F(2.273, 75.004)=1.938, p=.145 | F(2.073, 68.393)=.198, p=.828 | F(3.096, 102.153)=.700, p=.559 |
| *Time*Site*Mk* | F(6.18, 101.965)=.649, p=.695 | F(6.193, 106.99)=.094, p=.997 | F(5.066, 83.594)=1.347 p=.252 | F(4.546, 75.004)=.255, p=.924 | F(4.145, 68.393)=.231, p=.925 | F(6.191, 102.153)=.412, p=.874 |
| *Time*Site*Stimul*Mk* | F(6.18, 101.965)=.968, p=.452 | F(6.193, 106.99)=.204, p=.977 | F(5.066, 83.594)=.517 p=.765 | F(4.546, 75.004)=.141, p=.976 | F(4.145, 68.393)=.369, p=.836 | F(6.191, 102.153)=.791, p=.583 |
|  |  | | | | | |
| *Stimul* | F(1, 33)=.895, p=.351 | F(1, 33)=.113, p=.739 | F(1, 33)=6.264, p=.017 | F(1, 33)=7.826, p=.009 | F(1, 33)=.016, p=.902 | F(1, 33)=13.427, p=.001 |
| *Site* | F(2, 33)=.778, p=.468 | F(2, 33)=1.258, p=.297 | F(2, 33)=2.425, p=.104 | F(2, 33)=.597, p=.557 | F(2, 33)=.676, p=.516 | F(2, 33)=.172, p=.842 |
| *Mk* | F(1, 33)=.108, p=.745 | F(1, 33)=1.101, p=.302 | F(1, 33)=2.742, p=.107 | F(1, 33)=3.854, p=.058 | F(1, 33)=.023, p=.880 | F(1, 33)=3.977, p=.054 |
| *Stimul*Site* | F(2, 33)=.516, p=.602 | F(2, 33)=.531, p=.593 | F(2, 33)=.098, p=.907 | F(2, 33)=.318, p=.730 | F(2, 33)=.584, p=.563 | F(2, 33)=.688, p=.510 |
| *Stimul*Mk* | F(1, 33)=2.128, p=.154 | F(1, 33)=.006, p=.941 | F(1, 33)=.826, p=.370 | F(1, 33)=1.092, p=.304 | F(1, 33)=.080, p=.780 | F(1, 33)=2.969, p=.094 |
| *Site*Mk* | F(2, 33)=.293, p=.748 | F(2, 33)=.202, p=.818 | F(2, 33)=4.355, p=.021 | F(2, 33)=.224, p=.800 | F(2, 33)=.025, p=.975 | F(2, 33)=.683, p=.512 |
| *Stimul*Site*Mk* | F(2, 33)=.987, p=.383 | F(2, 33)=.158 p=.854 | F(2, 33)=.443, p=.646 | F(2, 33)=.013, p=.987 | F(2, 33)=.156, p=.856 | F(2, 33)=1.313, p=.283 |
