# Supplementary material for: iTBS-Induced LTP-Like Plasticity Parallels Oscillatory Activity Changes in the Primary Sensory and Motor Areas of Macaque Monkeys
Source: PLoS One. 2014 Nov 10;9(11):e112504. doi: 10.1371/journal.pone.0112504 (PMC4226540; doi:10.1371/journal.pone.0112504)
Supplement: Table S4 — Correlation coefficient between SEP N10 amplitude, band power and coefficient of variation Cv for each Site (M1, S1, M1–S1). (DOC) [file pone.0112504.s004.doc]

Table S4. Correlation coefficient between SEP N_10_ amplitude, band power and coefficient of variation Cv for each Site (M_1_, S_1_, M_1_-S_1_). Bonferroni corrected P values < .008 (values in bold) were considered significant.

|  |  | δ (1-4 Hz) | | θ (5-7 Hz) | | α (8-12 Hz) | | β (13-26 Hz) | | low γ (27-45 Hz) | | high γ (55-90 Hz) | |
| --- | --- | --- | --- | --- | --- | --- | --- | --- | --- | --- | --- | --- | --- |
|  |  | r | p | r | p | r | p | r | p | r | p | r | p |
| *Power - Cv* | *M_1_* | 0.503 | 0.387 | -0.324 | 0.595 | 0.554 | 0.333 | 0.898 | 0.039 | 0.809 | 0.097 | 0.889 | 0.043 |
|  | *S_1_* | -0.547 | 0.340 | 0.707 | 0.181 | 0.960 | 0.010 | **0.979** | **0.004** | 0.642 | 0.243 | **0.974** | **0.005** |
|  | *M_1_-S_1_* | 0.112 | 0.858 | -0.335 | 0.582 | 0.678 | 0.209 | 0.921 | 0.026 | 0.778 | 0.121 | **0.993** | **0.001** |
|  | |  | | | | | | | | | | | |
| *Power - N10* | *M_1_* | 0.790 | 0.112 | 0.007 | 0.991 | -0.010 | 0.987 | 0.152 | 0.807 | 0.064 | 0.919 | 0.801 | 0.103 |
|  | *S_1_* | 0.776 | 0.123 | 0.004 | 0.995 | 0.012 | 0.985 | 0.160 | 0.797 | 0.060 | 0.924 | 0.787 | 0.114 |
|  | *M_1_-S_1_* | 0.786 | 0.115 | 0.008 | 0.990 | 0.004 | 0.995 | 0.157 | 0.800 | 0.068 | 0.913 | 0.799 | 0.105 |
|  | |  | | | | | | | | | | | |
| *Cv- N10* | *M_1_* | 0.887 | 0.045 | 0.878 | 0.050 | 0.500 | 0.391 | 0.361 | 0.551 | 0.268 | 0.663 | 0.753 | 0.142 |
|  | *S_1_* | 0.097 | 0.877 | 0.229 | 0.710 | 0.144 | 0.818 | 0.278 | 0.650 | 0.520 | 0.370 | 0.856 | 0.064 |
|  | *M_1_-S_1_* | 0.690 | 0.198 | 0.857 | 0.063 | 0.424 | 0.477 | 0.322 | 0.597 | 0.334 | 0.583 | 0.851 | 0.067 |
